# I got frightened and felt strange. I even cried a lot after the diagnosis; the experiences on the screening and management of gestational diabetes mellitus among diagnosed women

**DOI:** 10.1186/s13104-023-06494-w

**Published:** 2023-09-29

**Authors:** Abdulai Abubakari, Mohammed Bukari, Hawa Malechi, Humphrey Garti, Faith Agbozo

**Affiliations:** 1 Department of Global and International Health, School of Public Health, University for Development Studies, Tamale, Ghana; 2 Department of Nutritional Sciences, School of Allied Health Sciences, University for Development Studies, Tamale, Ghana; 3 Department of Social and Behavioral Change, School of Public Health, University for Development Studies, Tamale, Ghana; 4 Department of Obstetrics and Gynaecology, Tamale Teaching Hospital, P.O. Box TL 16, Tamale, Ghana; 5 Department of Obstetrics and Gynaecology, School of Medicine, University for Development Studies, Tamale, Ghana; 6 Heidelberg Institute of Global Health, Heidelberg University, 69120 Heidelberg, Germany; 7 Department of Family and Community Health, Fred N. Binka School of Public Health, University of Allied Health Sciences, Private Mail Box 31, Ho, Ghana

**Keywords:** Gestational diabetes, Screening and management, Pregnancy, Emotions, Postpartum period, Ghana

## Abstract

**Introduction:**

Understanding the experiences of women diagnosed with Gestational Diabetes Mellitus (GDM) can improve screening, management, and postpartum care. Therefore, this study sought to investigate experiences on the screening and management of GDM among diagnosed women.

**Methods:**

This was a facility-based explorative qualitative design among five purposively sampled women diagnosed with GDM who were receiving care from healthcare professionals. Women were asked about their reaction to being diagnosed with GDM, their experiences with care, training, self-monitoring, and challenges with the management of GDM, and data obtained were analysed using thematic analysis.

**Results:**

Based on the thematic analysis, three main themes and ten sub-themes were generated. They were emotional experience (prior information on GDM before being diagnosed, and feelings about the diagnosis and blood glucose measurement), information source and care experience (source of information on healthy diet, training on blood glucose measurement, experiences with follow-up, and general impressions on GDM care), and dietary and lifestyle experience ( perceptions on dietary approaches, difficulties in getting and adhering to dietary and lifestyle guidelines, alternative treatment methods patronized, and effectiveness of dietary and lifestyle approaches).

**Conclusion:**

The themes generated had psycho-emotional underpinning, and underscores the importance of psychotherapy when disclosing disease status and initiating medical care. The findings of this study could be important for the optimisation of GDM care and services for affected women.

**Supplementary Information:**

The online version contains supplementary material available at 10.1186/s13104-023-06494-w.

## Introduction

Diabetes that is non-existent prior to pregnancy but which is triggered and develops during pregnancy is referred to as gestational diabetes mellitus (GDM). Agbozo and colleagues used different diagnostics criteria per fasting blood glucose and oral glucose tolerance cut-offs among 446 singleton pregnancies without pre-existing diabetes, the prevalence of GDM ranged from 8.3 to 23.8% and 4.4 -14.3% respectively [1]. Although Ghana has a national policy for the prevention and control of non-communicable diseases, emphasis is largely on cardiovascular diseases, cancers, chronic respiratory diseases and type II diabetes [2].

Meanwhile, a study by Han et al. (2015) demonstrated that despite majority of affected women viewing lifestyle control as crucial, GDM could still induce anxiety; an essential enabler for women to make desired lifestyle changes is consideration for the maternal and foetal health implications, while the main barrier is lack of family support [3]. Previous studies investigating the experiences of women with GDM found that negative feelings such as fear, shock, being upset or worried were more frequently mentioned [4–7]. Similar emotions of fear, shock and anxiety experienced by women diagnosed with GDM have been described in Australia, underscoring the emotional impact of diagnosis and management [8]. Women have been reported to encounter several obstacles in the self-management of GDM [5]; these included physical and social constraints, difficulty grasping the necessity of diet control, and regularly monitoring blood sugar levels within a short duration while still processing the diagnosis’s shock. They concluded that women from low socio-economic backgrounds struggle to understand and follow GDM dietary guidelines and exercise recommendations in the management of their conditions [5].

Many women lost personal control, as they were uninformed of the risk of developing active diabetes in the future [9]. In a metasynthesis, women were driven to control hyperglycemia by adhering to dietary and exercise recommendations to reclaim control and improve both maternal and foetal health outcomes; they concluded that health promotion materials that depict the varying experiences of GDM could prepare women, particularly at the time of diagnosis, on how to manage and control the condition [10]. Another study among immigrant women diagnosed with GDM reported positive care experiences, and the health education at the Diabetes in Pregnancy clinic helped them realise the value of adopting a healthy diet and lifestyle adjustments for their long-term health [11].

Additionally, Parsons and colleagues suggested that help from close relatives and healthcare professionals was essential for balancing and managing daily life [9]. Contrarily, stress, and inadequate support made the adaptation process more difficult [12]. An integrative review on education and intervention programs for GDM management showed that interventions such as consuming low-glycemic-index diets and exercising are effective at lowering maternal blood glucose levels and insulin needs during pregnancy [13]. Understanding the experiences of women diagnosed with GDM would be essential for improving screening and management services given to women with GDM. Yet, studies on the experiences of women diagnosed with GDM in northern Ghana are limited. Therefore, this study sought to investigate the experiences regarding screening and management among women in northern Ghana diagnosed with GDM.

## Methods

### Study area

Tamale, the capital of the Northern region of Ghana, served as the study’s location. Tamale has a population of 2,479,461 according to the 2020 population and housing census. Three of the region’s largest hospitals were chosen for the study: Tamale Teaching Hospital (TTH), Tamale Central Hospital (TCH), and Tamale West Hospital (TWH). According to the 2010 population census [13], total fertility rate of the Tamale metropolis is 2.8; its crude birth rate is 21.2/1000 and among women aged 15 to 49 and the general fertility rate is 79.9/1000.

### Study population

The study population was pregnant women diagnosed to have GDM and were being managed by healthcare professionals (HCPs).

### Study design

The study was a facility-based explorative study designed to explore the experiences of women diagnosed with GDM on the screening and management in northern Ghana using the phenomenological approach.

### Sampling techniques

Five study participants (diagnosed to have GDM) were selected using purposive sampling and data saturation was attained. Only pregnant women diagnosed with GDM and being managed by healthcare professionals were included in this study. As part of the diagnosis and management pathway in the major hospitals (TTH, TWH, and TCH) from which participants were selected, diagnosed women are asked to see an obstetrician and a dietician/nutritionist. Hence, for pregnant women to be considered for the purposive sampling, only those who used the services of an obstetrician and a dietician/nutritionist after the GDM diagnosis were invited to participate in this study. Three participants were recruited from the TTH because it is a tertiary facility and receives referrals from the other major facilities within the region; one participant each were recruited from TWH and TCH.

### Data collection

Data were obtained through five in-depth interviews with diagnosed women. Each interview was led by a facilitator, and a note taker was present to take notes while conversations were recorded. Interview guides were designed to elicit information on experiences on the screening and management of GDM (See supplementary material). Experiences on GDM diagnosis and care covered pregnant women’s reaction when first diagnosed with gestational diabetes, experience with management and care, health education, self-monitoring, and challenges with the management [14].

### Rigor and reflexivity

To improve the trustworthiness of our study, we addressed issues relating credibility, transferability, dependability, and confirmability as previously documented by Lincoln and Guba [15]. To establish the study’s credibility, study participants were given pre-publication copies and requested to provide input on the accuracy of the data. In terms of transferability, we supplied information about the study area, participant background information, and rich text of participants’ experiences. We engaged in peer-debriefing and member checking throughout the research process to ensure dependability and confirmability. Throughout the study, we kept an audit trail and engaged in regular reflexivity and bracketing.

We acknowledge that our roles as academics and healthcare professionals have influenced our ideas on how diagnosed women are screened for and treated for GDM. In order to reduce any bias we might have introduced into the study, we regularly engaged in bracketing. We recognised the existence of social dynamics and power structures. We made efforts to foster an atmosphere that allowed study participants to openly voice their opinions while resolving any power disparities that arose during interviews. We developed ethical and respectful interactions with the study participants by drawing on our Ghanaian identities.

### Data analysis

In-depth interviews were recorded, organised and transcribed verbatim, the themes were generated at the semantic level since focus was on the surface meanings of the data, using Braun and Clarke’s six steps for thematic analysis. The interviewers and data analysers were different people. After becoming familiar with the data through repeated reviews, initial codes were generated for the data and themes subsequently identified, which were then reviewed and further explained [16].

## Results

### Profiles of women diagnosed with GDM

Five women with a diagnosis of GDM were interviewed using an in-depth interview guide, the lowest age of the study participants was 32 years old, and the highest age was 45 years old. All of them were married, and mainly Christian with at least a tertiary degree as well as being gainfully employed (Table 1).

**Table 1:** Participant profiles of women diagnosed with GDM

Age	30–40	30–40	30–40	30–40	40–50
**Marital status**	Married	Married	Married	Married	Married
**Ethnicity**	Gonja	Frafra	Kasena	Frafra	Kasena
**Religion**	Muslim	Christian	Christian	Christian	Christian
**Education**	Primary	Degree	Masters’ degree	Degree	Degree
**Occupation**	Trader	Nurse	Teacher	Teacher	Civil servant

### Main themes on screening and management of GDM among diagnosed women

Based on the thematic analysis, three main themes and ten sub-themes were generated (Table 2). They were emotional experience (prior information on GDM before being diagnosed, and feelings about the diagnosis and blood glucose measurement), information source and care experience (source of information on healthy diet, training on blood glucose measurement, experiences with follow-up, and general impressions on GDM care), and dietary and lifestyle experience ( perceptions on dietary approaches, difficulties in getting and adhering to dietary and lifestyle guidelines, alternative treatment methods patronised, and effectiveness of dietary and lifestyle approaches).

**Table 2:** Themes and sub-themes generated on screening and management experiences of women diagnosed with GDM

Theme (s)	Sub-theme (s)
**Emotional experience**	Prior information on GDM before being diagnosed
	Feelings about the diagnosis and blood glucose measurement
**Information source and care experience**	Source of information on healthy diet
	Training on self-monitoring of blood glucose measurement
	Experiences with follow-up
	General impressions on GDM management
**Dietary and lifestyle experience**	Perceptions on dietary approaches
	Alternative treatment methods patronised
	Difficulties in getting and adhering to dietary and lifestyle guidelines
	Effectiveness of dietary and lifestyle approaches

### Emotional experience

#### Information on GDM prior to diagnosis

All the study participants had heard about GDM before they were diagnosed. They explained that it occurs when the body is not able to control the sugar in pregnancy and may or may not go after pregnancy. One of them added that it’s due to the food eaten as a result of cravings during pregnancy, and another added that family history is a risk factor. One of them indicated that she had never heard of GDM until she was diagnosed of it. She iterated: “This *is the first time I heard about it here at TTH.*” (Participant # 4).

*“Yes, I have heard about it. It mostly happens to pregnant women. Family history is a risk factor of GDM. It sometimes goes after pregnancy but other times too it does not.”* (Participant # 2).

*“Yes, I suspected I had it during my first pregnancy. During pregnancy some women develop diabetes due to the food they eat as a result of pregnancy cravings.”* (Participant # 1).

#### Feeling about the diagnosis and blood glucose measurement

When asked how they felt about the diagnosis, they were all disturbed as some were scared, worried, frightened, or even confused and sad.

One of them said *“Been diagnosed with GDM got me scared for some days. But I believe that if managed properly, I will be fine.”* (Participant # 4)

Another revealed *“I got frightened and felt strange. I even cried a lot after diagnosis.”* (Participant # 1).

Another confessed “*I became a little scared after diagnosis. Nobody wants to hear bad news more especially during pregnancy, but knowing my condition has indeed helped a lot.”* (Participant # 3).

On their reactions regarding the need to measure their blood glucose, two of them expressed uneasiness, two did not have the devices at home, and relied on health facilities while one of them was happy about it.

“*It is not easy to be pricking my finger anytime I want to check my blood sugar level.*” (Participant #4)

*“I was worried and anxious.”* (Participant # 5).

### Source of information and care experience

#### Source of information on healthy diet

On the most important source of advice on a healthy diet, one of them mentioned a women’s support group, while others mentioned their obstetricians, their husbands, their dieticians or nutritionist, or even the internet as the most important resource for advice on a healthy diet. For example one of them said:

*“My husband mostly reminds me about taking healthy diets and I sometimes use the internet to confirm my knowledge on a healthy diet.”* (Participant # 1).

#### Training on self-monitoring of blood glucose

On how they became proficient in self-monitoring of blood glucose, and how they found the training, a couple of them confirmed being trained by either their doctor or by their husband. Below is an excerpt from one of them.

*“My husband read the manual and later got training from a health care provider. It then became easier for my husband to train me.”* (Participant # 1).

#### Experiences with follow-up

All the women confirmed that their healthcare providers followed-up on them, however, only one of them disclosed being referred to a dietician after being diagnosed of GDM.

One of them said “*I lost my baby during my first pregnancy. For this present pregnancy, the doctor checks my blood sugar routinely.”* (Participant # 4).

Another confirmed: *“Yes, I was followed up on every two weeks by the obstetrician.”*(Participant # 1).

#### General impressions on GDM management

Those who were able to self-monitor their blood glucose expressed varied feelings about how it was working for them, one suggested that it could be expensive, while another expressed satisfaction with getting to monitor their blood sugar.

*“It is very helpful, because it helps you to monitor your blood sugar levels.”* (Participant # 1).

*“It is challenging because it demands self-discipline and can be expensive as well.”* (Participant # 5).

On their experiences of care-coordination and collaboration among different healthcare professionals involved in GDM care, they had varied impressions. One of them stated that she observed no care and coordination at public facilities while most of them were satisfied with it. Below is an excerpt from one of them.

*“The coordination of the care between the doctor and dietician was okay. The midwives were involved in taking and monitoring vitals.”* (Participant # 5).

All of them were satisfied with how their GDM were managed, and one even expressed a feeling of happiness for the early diagnosis. They suggested that in other to improve the quality of service during management of GDM, blood sugar should be regularly checked for early detection and management, while employing innovative ways to improve compliance to dietary and lifestyle approaches. They added that information on GDM preventive measures should be provided at antenatal care.

*“So far, I am okay with the management approach of GDM.”* (Participant # 1).

*“It is okay and I am satisfied with the current care.”* (Participant # 3).

*“It has been satisfactory”* (Participant # 5).

When asked how healthcare providers could deliver information about diet and self-monitoring blood glucose to them, they suggested that information about GDM should be put on flyers in the form of pictures for easier communication among pregnant women who come to the facilities, they also suggested that diet plan should include local foods, and that they should be given more education on GDM.

*“Health professionals should speak to the pregnant women when they come to the facilities. Information about GDM should be put on flyers in the form of pictures for easier communication among pregnant women who come to the facilities.”* (Participant # 4).

### Dietary and lifestyle experience

#### Perceptions on dietary advice received during GDM care

Four of the women were satisfied with the dietary advice they received during GDM care, one of them said the advice was difficult to follow due to pregnancy cravings.

*“It was difficult to follow the advice due to cravings for carbohydrates foods.”* (Participant # 3).

*“I am satisfied with the dietary advice. I was advised to consume wheat and foods rich in fiber. Also, to drink water every 30 minutes per day. I did not face any challenge in adhering to the advice.”* (Participant # 4).

*“I can feel that there is improvement in my condition after the dietary advice. I complied with the consumption of green leafy vegetables, minimized my sugar intake, drank a lot of water, and also had a walk sometimes.”* (Participant # 1).

On how they felt about getting information about diet from different sources, four of them said they were happy about it, while one of them indicated that she did not use other sources aside from the obstetrician.

“*I did not seek information from other people apart from visiting the gynecologist*” (Participant # 2).

*“It was helpful especially with Dr. [name withheld]. She asked meaningful questions. Also experiences from support groups enhanced my confidence to follow the guidelines”* (Participant # 3).

#### Difficulties in getting information and adhering to dietary and lifestyle guidelines

Almost all (four) the women said they did not experience any difficulty in receiving information from different healthcare professionals.

One said *“No, it was easier getting the information from my husband and I am able to abide by the guidelines due to self-discipline*.” (Participant # 2).

All the women expressed some level of difficulty in following dietary and lifestyle guidelines with pregnancy cravings being a barrier. Their various responses have been captured below.

*“It is challenging due to pregnancy craving but I am able to manage my way through.”* (Participant # 3).

*“I find it difficult to follow the dietary guidelines and also it is difficult to walk”* (Participant # 4).

*“Following the guidelines is not difficult. I was even asked to take red millet. But sometimes it difficult to get fruits such as watermelon and vegetables. The main issue is the affordability and the accessibility of the fruits and vegetables.”* (Participant # 1).

*“I do face some difficulties following dietary and lifestyle guidelines. This includes challenges in following the eating timeline due to work. Also, pregnancy cravings were also a problem.”* (Participant # 5).

#### Alternative treatments patronised

Four of the women said they were not using alternative treatments other than the hospital treatments. One person said “I drink boiled “langrindoo” (*Aeschynome afraspera*),“pringkpang” (*Imperata cylindrica*) leaves and lemon (*Citrus limon*).” (Participant # 1).

#### Effectiveness of dietary and lifestyle approaches

All the women perceived the dietary and lifestyle approaches to be effective in helping to manage their condition.

*“Yes, it is very effective. I observed that my blood sugar reading was very high in the first week. But after following the guidelines for some days it went down completely.”* (Participant # 2).

*“Yes, I think they are effective and I have seen improvement after following the guidelines.”* (Participant # 3).

*“Yes, I do perceive them to be helpful and effective. I felt an improvement after following the dietary and lifestyle guidelines.”* (Participant # 1).

## Discussion

This study sought to explore experiences on the screening and management of GDM among diagnosed women. Three main themes were generated: emotional experience, information source and care experience, and dietary and lifestyle experience. The study found that most women were disturbed after being diagnosed to have GDM. The most important sources of information on GDM were the healthcare professionals (HCPs) specifically obstetricians, dieticians or nutritionists, husbands, support groups, and the internet. Women were generally satisfied with GDM management, particularly with the dietary advice and the diverse sources of information; eventhough some expressed difficulty in compliance as a result of pregnancy-induced food cravings and food unavailability. On how they felt about self-monitoring of blood glucose, they expressed concerns regarding cost and the pain associated with having to prick their fingers.

One of the themes generated on the screening and management experiences of women diagnosed with GDM was emotional experience. This current study showed that women were disturbed when diagnosed with GDM. They expressed emotions of fear, sadness, confusion, and worry. A study by Helmerson and colleagues also reported that women were shocked, worried or even blamed themselves when they were diagnosed with GDM [14]. In addition, previous studies investigating the experiences of women with GDM reported that negative feelings such as being upset, fear, shock, or worries were more frequently mentioned [4–7]. In the antenatal care (ANC) setups in the study area, women are often given health talks, especially on non-communicable diseases, so it makes sense that women with prior information on GDM would be disturbed when they are diagnosed to have GDM; particularly those aware of the maternal and foetal implications of GDM. Unsurprisingly, anxiety over maternal and foetal health outcomes have been documented to be potential enablers for compliance with dietary and lifestyle modification regimens when controlling or managing GDM [3, 10].

The second theme was information source and care experience. The most important source of information for women on GDM were from obstetricians, dieticians or nutritionists, husbands, mother support groups, and the internet; and women were satisfied with receiving information from varied sources. Interestingly, another study reported that women expected their general practitioners to be the best sources of information while citing the internet and support groups as other ideal sources for information on GDM [17]. A study by Edwards and colleagues also reported that women used social media to meet their information needs, which is in line with the findings of the current study where some mentioned the internet as an important source of information [18].

On care experience, women were generally satisfied with GDM care, as they expressed satisfaction with receiving information from different sources on dietary and lifestyle approaches. Sayakhot and colleague also reported that women were satisfied with how they were informed about their GDM diagnosis [17]. We observed similar findings, but our participants suggested that other innovative approaches should be considered to enhance compliance to dietary and lifestyle regimens.

With regards to the theme on dietary and lifestyle experiences, women perceived dietary and lifestyle approaches for reducing GDM to be effective. One of the dietary regimens used in the management of GDM as reported in the current study was the DASH diet; this dietary regimen has been reported to be effective in improving maternal outcomes and glycaemic control [19–21]. A Cochrane review in 2017 reported moderate quality evidence of a combined intervention of diet and exercise as compared to standard care in reducing the risk of GDM [22]. The Norwegian Directorate of Health has also recommended that women should be given dietary advice and trained on self-monitoring of blood glucose [23]. Currently, no guidelines exists for managing GDM in Ghana, HCPs use other guidelines with adaptations to the Ghanaian context, which are not very different from the practices recommended by the Norwegian guidelines [23]. For instance, while giving women dietary and lifestyle advice, those who can afford glucometres are also trained on self-monitoring of blood glucose. Even those who could afford or had access to glucometres acknowledged that self-monitoring of blood glucose was expensive. This highlights the economic burden GDM places on affected individuals and could be a major obstacle in effective treatment or management of GDM. Hence, the relevant stakeholders should consider making glucometres and test strips more accessible and affordable, as this would enhance treatment or management of GDM.

Despite women perceiving dietary and lifestyle approaches to be effective in managing GDM, they admitted some difficulties in adhering to dietary and lifestyle guidelines. Some of the challenges they mentioned were pregnancy cravings, reluctance to exercise, scarcity of fruits and vegetables, and workload. It has been reported that appetite and perceived ability to control cravings may have an effect on gestational weight gain [24], which is associated with GDM [25–27].

### Strengths and limitations

The strength of this study is that it provides insights on GDM screening and management among women diagnosed in northern Ghana. This study had some limitations as well. Ghana runs a three-tiered health system and participants were selected from secondary and tertiary level health facilities; hence the findings of this study may not be representative of the views of those who use primary level care facilities. However, it is worth noting that diagnosis of GDM is mainly at the secondary and tertiary level health facilities, as all suspected cases at the primary care levels are usually referred to them. Another major limitation of this study is that almost all the sampled participants were married, gainfully employed with a tertiary degree and were Christians, which does not reflect the current demographic characteristics of people from northern Ghana. This could be because only women who patronised specialised care from nutritionist/dieticians and an obstetrician were included in this study. Nonetheless, we are confident that the views from the current study provide key insights that could be essential for the optimisation of GDM care and services among affected women in northern Ghana. Also, the researcher’s reflexivity during analysis and interpretation of findings could have affected the findings. To address this, other authors were actively involved in the research process to minimize this bias.

## Conclusion

This study sought to explore the experiences of women diagnosed with GDM in northern Ghana on its screening and management. Three major themes were generated on screening and management experiences for women diagnosed with GDM; emotional experience, information source and care experience, and dietary and lifestyle experience. The themes generated from this study have psycho-emotional underpinning underscoring the importance of psychotherapy when disclosing disease status and initiating medical care. The findings of this study could be important for the optimisation of GDM care and services for affected women.

### Electronic supplementary material

Below is the link to the electronic supplementary material.

Supplementary Material 1

## Data Availability

The dataset supporting the findings of this study could be obtained from the corresponding author upon reasonable request.

## Abbreviations

HCPs: Healthcare professionals
GDM: Gestational diabetes mellitus
ANC: Antenatal care
DASH: Dietary Approaches to Stop Hypertension
