# Supplementary material for: I got frightened and felt strange. I even cried a lot after the diagnosis; the experiences on the screening and management of gestational diabetes mellitus among diagnosed women
Source: BMC Res Notes. 2023 Sep 29;16:236. doi: 10.1186/s13104-023-06494-w (PMC10540323; doi:10.1186/s13104-023-06494-w)
Supplement: Supplementary file 1 — Supplementary Material 1 [file 13104_2023_6494_MOESM1_ESM.docx]

**I got frightened and felt strange. I even cried a lot after the diagnosis; the experiences on the screening and management of gestational diabetes mellitus among diagnosed women**

**Socio-demographic characteristics**

Age:

Marital status:

Ethnicity:

Religion:

Educational status;

Occupation:

**Interview guide:**

Have you heard about gestational diabetes before you were diagnosed? What have you heard about it?

How did you feel about the diagnosis?

Can you tell me something about your general experiences with follow up of your gestational diabetes (have you been followed up by your GP/midwife or/and the outpatient clinic at the hospital?)

**Main topics and probing questions:**

- Experiences of getting information about a healthy diet and lifestyle

What do you think about the dietary advice you have received during your GDM care?

Who/what was your most important source for advice about a healthy diet?

How did you feel getting information about diet from different health care professionals?

Did you experience any difficulties in getting information about diet from different health care professionals?

Was there something you missed when receiving information about diet?

Aside hospital treatments, are they any alternative treatments that you patronize (herbs/ special foods)?

Do you have difficulties following dietary and lifestyle guidelines?

Do you perceive the dietary and lifestyle approaches to be effective in the management of your condition?

Are they any religious or spiritual reasons/ factors that should be catered for in the management of gestational diabetes?

- **Experiences in getting training in self-monitoring of blood glucose**

What did you think when you got to know that you had to measure your own blood glucose?

Who trained you in self-monitoring of blood glucose? How did you experience the training?

How do you feel the self-monitoring of blood glucose is working?

Can you tell me something about your experiences in getting training from different health care professionals?

- **Women’s informational needs about diet and self-management of blood glucose**

In what way do you think health care professionals could in best way adapt information about diet and self-monitoring blood glucose to you as a pregnant with gestational diabetes?

- Experiences of care-coordination and collaboration of different health care professionals involved in GDM care

How did you experience the collaboration between different health care professionals in providing you with information about diet and training in self-monitoring of blood glucose?

What would you say about how the GDM is managed?

What can be done to improve the quality of service given to you in the management of GDM?
